# Antigingivitis efficacy of a sodium bicarbonate toothpaste: Pooled analysis

**DOI:** 10.1111/idh.12626

**Published:** 2022-10-05

**Authors:** Charles R. Parkinson, Andrew Butler, Martin R. Ling

**Affiliations:** ^1^ Haleon Weybridge UK

**Keywords:** gingivitis, plaque, pooled analysis, sodium bicarbonate, toothpaste

## Abstract

**Objective:**

The objective of this study was to investigate the antigingivitis and antiplaque treatment effect of a toothpaste containing 67% w/w sodium bicarbonate, at the individual tooth site, tooth region and whole mouth (overall) level, by way of a pooled analysis of data from similarly designed clinical trials.

**Methods:**

Six randomized controlled GSKCH clinical trials, 12–24 weeks in duration, were selected based on pre‐specified criteria which included access to patient level data, pre‐treatment dental prophylaxis, >20 bleeding sites and mild–moderate gingivitis at screening and use of 67% sodium bicarbonate toothpaste and non‐sodium bicarbonate (regular) toothpaste (negative control) for ≥4 weeks. Efficacy outcomes comprised plaque index (TPI), modified gingival index (MGI) and gingival bleeding (bleeding index (BI), number of bleeding sites (BS)). Treatment comparisons were made using ANCOVA for whole mouth (overall) scores and by tooth site region (facial overall, lingual overall; margin/body overall, facial margin/body, lingual margin/body; papillae/interproximal overall, facial papillae/interproximal, lingual papillae/interproximal). Pooled data for BI, MGI, and TPI at individual tooth sites was plotted as a mouthmap to summarize treatment response (change from baseline) by tooth site, at the 24‐week timepoint.

**Results:**

For all measures, whole mouth and for all tooth regions at all post‐treatment timepoints, significant (*p* < 0.001) differences in favour of the 67% sodium bicarbonate toothpaste compared with control were observed. At the 24‐week timepoint, facial regions demonstrated greater improvements than lingual regions, with the greatest between treatment improvement seen for the facial‐papillae regions for bleeding (BS/BI), facial‐margin regions for MGI and facial‐body regions for TPI. All individual tooth sites demonstrated numerically greater reductions from baseline for the 67% sodium bicarbonate toothpaste than the control toothpaste following 24 weeks use, with the greatest improvements (change from baseline) seen for posterior and papillae tooth sites for bleeding, margin tooth sites for MGI and body tooth sites for TPI.

**Conclusion:**

This pooled analysis of patient level‐data, limited to GSKCH long‐term gingivitis clinical studies, demonstrates that twice daily use of a 67% sodium bicarbonate toothpaste effectively removes plaque from all tooth sites, and results in clinically significant improvements in measures of gingival health, overall and for all the tooth regions investigated, compared with a non‐sodium bicarbonate (regular) toothpaste following 24 weeks twice daily use.

## 1 INTRODUCTION

Gingivitis is an inflammatory response to the presence of dental plaque,^1^ which typically presents as redness, swelling (oedema), and/or bleeding of the gums at the gingival margin surrounding the tooth. Gingivitis is a reversible condition but if left untreated it can progress to the irreversible phase of periodontitis, where inflammation extends to the underlying tissues, periodontal ligament, and alveolar bone.^2^ The resulting loss of these structures can eventually lead to tooth loss through destruction of the periodontal tissues supporting the tooth.^2^

Dental plaque is a soft, sticky, colourless deposit of bacteria which collects on the teeth and along the gingival margin. It is a causative agent of gingivitis and periodontitis.^3^, ^4^ Gingivitis can be prevented and resolved through effective plaque control, primarily via mechanical plaque removal, i.e., toothbrushing.^4^, ^5^

Sodium bicarbonate is an ingredient added to toothpastes to aid plaque removal. It is hypothesized to physically displace plaque from the tooth surface (mechanical disruption by particles of sodium bicarbonate), and/or interfere with the adhesion characteristics of the biofilm leading to a reduction of biofilm structural integrity.^6^ Numerous clinical studies have been conducted evaluating the efficacy of 67% sodium bicarbonate toothpastes to reduce plaque and provide improvements in gingival health.^7^, ^8^, ^9^, ^10^, ^11^, ^12^, ^13^, ^14^, ^15^, ^16^ These studies generally report improvements in the measures of plaque and/or gingival health in favour of the 67% sodium bicarbonate toothpaste compared with a control toothpaste (non‐sodium bicarbonate toothpaste). In a meta‐analysis investigating the efficacy of sodium bicarbonate toothpastes in controlling plaque and gingivitis, Valkenburg et al.^17^ reported that sodium bicarbonate toothpastes showed ‘promising results with respect to plaque in single use studies’, however only a small improvement in gingival health (as measured by bleeding scores) for sodium bicarbonate toothpastes relative to a control product was reported. The analysis highlighted significant variability and uncertainly in the composition of the sodium bicarbonate toothpastes, where the quantity of sodium bicarbonate in toothpaste can range from 35% to 67% w/w. Sodium bicarbonate concentration in toothpaste has been suggested to have positive relationship for plaque removal efficiency, but a statistically significant relationships has not been observed.^6^

Single use clinical studies conducted specifically on 67% sodium bicarbonate toothpastes have reported that the greatest plaque removal advantage for sodium bicarbonate toothpastes, compared with non‐sodium bicarbonate toothpastes, is in the lingual interproximal areas.^14^, ^18^ It would therefore be valuable to explore the plaque control and gingival health treatment effect of 67% sodium bicarbonate toothpastes, overall and at the tooth region/tooth site level to interrogate the mode of action of 67% sodium bicarbonate toothpastes, and to explore whether the results from single‐use brushing studies translate to similar plaque control and improvements in gingival health in longer‐term (>4 week) gingival health clinical studies.

We (GSK Consumer Healthcare/Haleo [GCKCH]) have conducted and reported six long‐term (>4 week) clinical studies investigating improvements in plaque control and gingival health associated with pre‐existing gingivitis, for a 67% sodium bicarbonate toothpaste relative to a negative control toothpaste (regular toothpaste without sodium bicarbonate).^10^, ^11^, ^13^, ^15^, ^16^ These studies were of very similar design, following guidelines for the conduct of antigingivitis studies.^19^ They were conducted in three different countries (India, China and USA) and employed different examiners. A pooled analysis of these studies, with available patient‐level data, can provide reliable information (with reduced heterogeneity) on the treatment effect for 67% w/w sodium bicarbonate toothpastes compared with a non‐sodium bicarbonate toothpaste for the whole mouth, by tooth site region and at the individual tooth site level, and permit comment on the purported mode of action of sodium bicarbonate.

The aim of this study was therefore to provide a comprehensive overview of antigingivitis and plaque control treatment effect for 67% w/w sodium bicarbonate toothpastes compared with a negative control toothpaste, for whole mouth (overall) and by tooth site region (facial overall, lingual overall; margin/body overall, facial margin/body, lingual margin/body; papillae/interproximal overall, facial papillae/interproximal, lingual papillae/interproximal) and individual tooth site by way of a pooled analysis of similarly conducted clinical studies employing 67% w/w sodium bicarbonate toothpaste. The primary outcome was to be considered successful if there was a minimum statistically significant difference of 15% observed between the 67% w/w sodium bicarbonate toothpaste and the negative control [0% sodium bicarbonate toothpastes or regular marketed toothpastes] for the overall number of bleeding sites to infer on potential effectiveness and permit comment on the relative efficacy by tooth region.

## 2 MATERIALS AND METHODS

### 2.1 Pooled analysis study design

Six randomized, blinded, parallel‐group controlled clinical studies investigating a 67% w/w sodium bicarbonate‐containing toothpaste on measures of gingivitis and plaque accumulation were identified from our records (reported prior to 2018) for inclusion in the pooled‐analyses (13 studies were identified, and six met pre‐defined inclusion criteria). Inclusion of these studies in the pooled analysis was determined based on those meeting the pre‐specified criteria and in agreement with Preferred Reporting Items for Systematic Reviews and Meta‐Analyses (PRISMA) guidelines (Setting Inclusion Criteria to Avoid Bias in Selecting Studies). The criteria for the selection of studies were set a priori and based on criteria for establishing efficacy of antigingivitis of toothpastes (population, disease stage, clinical assessment measure[s], length of study, treatment procedures) and access to patient‐level data. Two reviewers assessed the studies for inclusion. The following pre‐specified criteria were employed: clinical studies conducted by GSKCH/Haleo allowing full access to study protocols, reports and patient level study data; inclusion of a pre‐treatment full‐mouth prophylaxis; subject eligibility (>20 bleeding sites and mild–moderate gingivitis at Screening); comparable indices of gingivitis or plaque accumulation, and a treatment consisting of a 67% w/w sodium bicarbonate toothpaste for at least 4 weeks. Details of all the studies identified and reason(s) for exclusion, along with the study location and study durations, salient procedural aspects and summary details of the participation criteria for subjects in the six studies are provided in supplementary data Tables S1 and S2. The trial was registered at clinicaltrials.gov (NCT03703245). Anonymized individual participant data and study documents can be requested for further research from www.clinicalstudydatarequest.com.

#### 2.1.1 Individual studies

All studies were single‐center, examiner‐blind, 2 or 3 treatments, parallel group, stratified, randomized, with a dental prophylaxis performed at Baseline (or at Screening) prior to start of randomized product use. The study design, selection of endpoints, and controls in these studies were similar (supplementary data Tables S1 and S2). Eligible subjects were randomly assigned to one of either a 67% w/w sodium bicarbonate toothpaste (or additionally 62% w/w sodium bicarbonate toothpaste where also investigated), or a negative control (non‐sodium bicarbonate toothpaste). Subjects used the allocated study treatment as part of twice‐daily toothbrushing for 6 weeks,^13^ 12 weeks^10^, ^11^ and 24 weeks.^15^, ^16^ For all studies, the examiner, study statistician and employees of GSKCH who may have influenced study outcomes were blinded to treatment allocations. The test products were supplied in plain tubes to help maintain blinding. The primary endpoints of the individual studies were gingival bleeding (bleeding site, BI at four sites) and inflammation (MGI at four sites) at the final time point measured. Newby et al.^11^ included use of the Gingival Index (GI) (at six sites), rather than MGI (four sites), as the index to measure visual signs of gingival inflammation. Discussed later (exclusion of data), it was not possible to include study Newby et al.^11^ for the pooled analysis for measures of gingival bleeding and inflammation, however, this study included plaque/TPI data on comparable tooth surfaces and therefore plaque/TPI data from Newby et al.^11^ was included in the pooled analyses.

A single population was defined for each study for all Safety, Baseline, and Demographic summaries, as well as for the analysis of efficacy measures. The Intent‐to‐Treat (ITT) Population consisted of all subjects who were randomized to treatment, who were dispensed study medication, and had at least one post‐baseline efficacy measure.

#### 2.1.2 Study products

All the clinical studies employed substantially equivalent 67% w/w sodium bicarbonate toothpastes; the Test sodium bicarbonate formulations only differed with respect to flavour and fluoride level (932 ppm to 1450 ppm fluoride, as sodium fluoride, according to local regulation under which the studies were conducted). The control formulations were either marketed regular fluoride toothpastes in the county where the study was conducted (Crest Cavity Protection for Jose et al.^16^ and Akwagyirama et al.,^15^ MacLeans FreshMint for Newby et al.^11^ or non‐marketed regular fluoride toothpastes manufactured by GSK Consumer Healthcare/Haleon Kakar et al.^10^; Lomax et al.^13^). These ‘non‐marketed’ formulations were versions of Aquafesh Fresh and Minty, without the coloured stripes.

### 2.2 Participants

The subject populations included male and non‐pregnant, non‐lactating female subjects at least 18 years of age with pre‐existing gingivitis (mild to moderate) as determined by an appropriately qualified clinical examiner.

### 2.3 Statistical analysis

Data for the studies included in this pooled analysis were anonymized prior to analyses. Individual subject data were used from the individual studies.

All efficacy variables were summarized by descriptive statistics (n, adjusted mean, standard error [SE]). Statistical analyses tested the null hypothesis of no difference between treatments versus the alternative that there is a difference between treatments. Two‐sided statistical significance testing (alpha level [*α*] = 0.05) comparing 67% sodium bicarbonate toothpaste to negative control toothpaste was performed for the primary and secondary endpoints using the analysis of covariance (ANCOVA) method of analysis with factors for treatment and study including the baseline value as a covariate and smoking status (yes/no) as a stratification factor. The statistical analysis software used was SAS Studio version 9.4. Analyses were based on the ITT Population.

Missing data were not imputed. Overall, there were very little missing data (all included studies had <10% missing data).

#### 2.3.1 Analysis periods

For the pooled data, analyses were performed at Week 6, Week 12, and Week 24 as determined by the time points included in the individual clinical studies.

#### 2.3.2 Exclusion of data from analysis

All data recorded were included where possible. If it was not possible to convert data from one clinical index to another then the most common index was used. Newby et al.^11^ was not included in the pooled analysis for measures of gingival inflammation as the GI index (measured at six tooth sites), is neither directly nor accurately comparable to the MGI nor the number of bleeding sites/BI (measured at four tooth sites). However, plaque/TPI data was included in the pooled analysis as it was on comparable tooth surfaces to the other studies.

#### 2.3.3 Demographic and baseline characteristics

Demographic and baseline characteristics summaries were produced on the ITT Population for individual studies and pooled data. No formal analysis of the demographic data was conducted; the characteristics were summarized by treatment groups. The summary of demographic characteristics included age, sex, race, and ethnicity (where available). The summary of baseline characteristics included stratification factor, baseline number of bleeding sites (low and high), and smoking status (smoker and non‐smoker).

#### 2.3.4 Efficacy analysis

Efficacy variables for each of the indices under investigation were ‘overall (whole mouth/region)’, ‘margin’, ‘papillae’ for number of bleeding sites, BI and MGI; ‘overall’, ‘body’, and ‘interproximal’ (distal and mesial) for TPI. Papillae and margin (number of bleeding sites, BI, and MGI) are considered analogous to interproximal (distal and mesial) and body sites (TPI), respectively. The specific teeth regions of interest included overall (whole mouth), lingual (all teeth lingual sites), and facial (all teeth facial sites).

#### 2.3.5 Primary efficacy endpoint

The primary efficacy analysis for this pooled analysis focused on the number of bleeding sites over 24 weeks as this is the longest time point in the individual studies included in the pooled analysis, and is considered to be a scientifically and clinically valid endpoint on which to infer on potential effectiveness of 67% sodium bicarbonate in improving gingival health. The outcome was to be considered successful if there was a minimum statistically significant difference of 15% observed between the 67% w/w sodium bicarbonate toothpaste and the negative control toothpaste for the number of bleeding sites to infer on potential effectiveness.^19^

The efficacy variable of number of bleeding sites/index, MGI and plaque (overall, margin/body, and papillae/interproximal) was summarized and analysed for each of the three overall tooth sites (overall, lingual, and facial). Each time point data were summarized (n, missing, mean, median, SD, SE, minimum, and maximum) for the above efficacy variables by treatment groups. This dataset included the adjusted mean and SE of each treatment group, adjusted means (SE) of the treatment difference between 67% sodium bicarbonate and 0% sodium bicarbonate (negative control), 95% CI, % difference, and *p*‐value.

The variables were summarized into a single dataset and a summary table by study and pooled for investigation of treatment effects in all areas. Mouth map plot of overall score for all tooth sites and for various time points were produced for variables BI, MGI, and TPI for pooled data.

Exploration of Country Effect: The overall efficacy variables were explored via tabulation by country and treatment groups only. This summary allowed exploration of potential cultural differences in measures/responses associated with measures of gingivitis and plaque accumulation in groups of subjects associated with the studies selected in this pooled analysis. No formal statistical analyses were performed for this. The descriptive summary (n, missing, mean, SD, SE, median, minimum, and maximum) was presented by country and treatment group for overall number of bleeding sites, BI, MGI, and TPI and for overall tooth sites.

## 3 RESULTS

### 3.1 Participants

A total of 2846 subjects were screened across all six studies (in three counties, India (three studies), USA (two studies) and China (one study)) [all subjects]. Of these, 1601 subjects were randomized ‐ 640 subjects in 67% sodium bicarbonate group and 641 subjects in Negative Control group (320 subjects were in a third treatment group ‐ 62% sodium bicarbonate). A total of 1474 (92.1%) subjects completed the studies. In the 67% sodium bicarbonate group, 57 subjects did not complete the study (four did not meet study criteria; one withdrew due to an adverse event; 31 were lost to follow‐up; five had protocol violations; 15 withdrew consent; one listed as ‘other’). In the Negative control group, 50 subjects did not complete the study (one withdrew due to an adverse event; 35 were lost to follow‐up; two had protocol violations; 10 withdrew consent; two listed as ‘other’).

#### 3.1.1 Demographic characteristics

A total of 908 subjects (59.2%) were female and 626 subjects (40.8%) were male across all 6 pooled studies [ITT population]. The majority of subjects (70.7%) were of Asian heritage, 22.2% of subjects were of White heritage. The majority of subjects (54.4%) were not of Hispanic or Latino ethnicity (Ethnicity was only captured in two studies). The mean (SD) age of subjects was 34.2 years (11.07 years) with a range of 18 to 79 years across all six studies.

#### 3.1.2 Baseline characteristics

Overall, 1000 (65.2%) subjects had a high number of bleeding sites (≥45) at baseline and the majority of subjects (91.2%) were non‐smokers across all 6 studies. A total of 929 (60.6%) subjects were non‐smokers with baseline number of bleeding sites ≥45; 71 (4.6%) were smokers with ≥45 bleeding sites; 470 (30.6%) were non‐smokers with <45 bleeding sites; 64 (4.2%) were smokers with <45 bleeding sites.

### 3.2 Efficacy

The primary endpoint (number of bleeding sites) and secondary efficacy endpoints (BI, MGI and TPI) for overall (whole mouth/region) and tooth site region are summarized in Tables 1 and 2, respectively. The between‐treatment differences (67% sodium bicarbonate vs negative control) in the adjusted mean for all measures for whole mouth (overall), at all timepoints were statistically significant (*p* < 0.0001) in favour of 67% sodium bicarbonate toothpaste, and demonstrated progressive improvements at each time point.

**TABLE 1:** Bleeding sites mean values and treatment differences overall and by tooth site region and visit (ITT population)

Tooth Site Region	Visit (week)	67% Sodium bicarbonate		Negative control		Comparison of 67% sodium bicarbonate with negative control in number of bleeding sites		
		*n*	Adjusted Mean^a^ (SE)	*n*	Adj mean^a^ (SE)	Adj mean diff (SE)	95% CI	% Diff
Overall	Baseline	498	56.4 (1.03)	505	56.0 (1.01)			
	6	497	25.55 (0.403)	505	36.32 (0.400)	−10.77 (0.562)*	(−11.87, −9.66)	−29.64
	12	410	18.19 (0.409)	422	30.52 (0.404)	−12.34 (0.569)*	(−13.45, −11.22)	−40.42
	24	222	15.93 (0.596)	228	30.77 (0.588)	−14.84 (0.837)*	(−16.48, −13.19)	−48.22
Overall lingual	Baseline	498	31.0 (0.54)	505	30.9 (0.51)			
	6	497	14.94 (0.259)	505	20.48 (0.257)	−5.54 (0.361)*	(−6.25, −4.83)	−27.07
	12	410	10.85 (0.262)	422	17.20 (0.259)	−6.35 (0.364)*	(−7.06, −5.63)	−36.93
	24	222	10.07 (0.365)	228	17.98 (0.360)	−7.91 (0.513)*	(−8.92, −6.90)	−43.99
Overall facial	Baseline	498	25.4 (0.55)	505	25.1 (0.55)			
	6	497	10.63 (0.220)	505	15.83 (0.218)	−5.20 (0.307)*	(−5.81, −4.60)	−32.87
	12	410	7.34 (0.219)	422	13.32 (0.217)	−5.98 (0.305)*	(−6.58, −5.38)	−44.88
	24	222	5.87 (0.343)	228	12.78 (0.339)	−6.90 (0.482)*	(−7.85, −5.96)	−54.03
							
Margin overall	Baseline	498	28.1 (0.60)	505	27.8 (0.60)			
	6	497	13.64 (0.230)	505	18.48 (0.228)	−4.85 (0.321)*	(−5.47, −4.22)	−26.22
	12	410	9.46 (0.227)	422	14.98 (0.224)	−5.53 (0.316)*	(−6.15, −4.91)	−36.89
	24	222	7.38 (0.349)	228	13.90 (0.344)	−6.52 (0.490)*	(−7.48, −5.56)	−46.92
Margin lingual	Baseline	498	16.2 (0.35)	505	16.2 (0.34)			
	6	497	8.49 (0.159)	505	10.95 (0.157)	−2.46 (0.221)*	(−2.89, −2.02)	−22.44
	12	410	5.97 (0.156)	422	8.87 (0.154)	−2.89 (0.217)*	(−3.32, −2.47)	−32.64
	24	222	4.64 (0.215)	228	7.99 (0.212)	−3.36 (0.302)*	(−3.95, −2.76)	−41.99
Margin facial	Baseline	498	11.9 (0.31)	505	11.7 (0.30)			
	6	497	5.15 (0.126)	505	7.51 (0.125)	−2.36 (0.176)	(−2.70, −2.01)	−31.40
	12	410	3.48 (0.124)	422	6.11 (0.122)	−2.62 (0.172)	(−2.96, −2.29)	−42.97
	24	222	2.75 (0.206)	228	5.89 (0.203)	−3.14 (0.290)	(−3.71, −2.57)	−53.28
							
Papillae overall	Baseline	498	28.3 (0.48)	505	28.2 (0.46)			
	6	497	11.93 (0.240)	505	17.82 (0.238)	−5.89 (0.335)*	(−6.55, −5.23)	−33.05
	12	410	8.73 (0.251)	422	15.53 (0.248)	−6.80 (0.349)*	(−7.48, −6.11)	−43.77
	24	222	8.56 (0.378)	228	16.86 (0.373)	−8.30 (0.531)*	(−9.34, −7.26)	−49.23
Papillae lingual	Baseline	498	14.8 (0.24)	505	14.7 (0.23)			
	6	497	6.46 (0.156)	505	9.53 (0.155)	−3.07 (0.218)*	(−3.50, −2.64)	−32.21
	12	410	4.88 (0.159)	422	8.33 (0.157)	−3.45 (0.221)*	(−3.89, −3.02)	−41.42
	24	222	5.41 (0.239)	228	10.01 (0.236)	−4.59 (0.336)*	(−5.25, −3.93)	−45.90
Papillae facial	Baseline	498	13.5 (0.29)	505	13.4 (0.29)			
	6	497	5.48 (0.143)	505	8.29 (0.142)	−2.81 (0.200)*	(−3.20, −2.42)	−33.92
	12	410	3.86 (0.142)	422	7.20 (0.141)	−3.34 (0.198)*	(−3.73, −2.95)	−46.43
	24	222	3.14 (0.214)	228	6.86 (0.211)	−3.71 (0.301)*	(−4.31, −3.12)	−54.16

*Note*: % Differ = 100* (Adjusted mean in 67% sodium bicarbonate – adjusted mean in negative)/(Adjusted mean in negative control).

Abbreviations: CI, confidence interval, % Diff, percentage difference, ITT, intent‐to‐treat, *n*, number of subjects, SE, standard error.

^a^ Unadjusted mean for baseline values.

* *p* < 0.0001.

**TABLE 2:** Treatment (% difference) by tooth site region, index and timepoint (ITT population)

		Comparison of 67% sodium bicarbonate with negative control toothpaste								
		Bleeding Index (BI)			Gingival Inflammation (MGI)			Plaque Index (TPI)		
Tooth Site Region	Visit (week)	Mean Diff (SE)	95% CI	% Diff	Mean Diff (SE)	95% CI	% Diff	Mean Diff (SE)	95% CI	% Diff
Overall (whole mouth)	6	−0.12* (0.006)	−0.13, −0.11	−30.88	−0.30* (0.020)	−0.34, −0.26	−15.43	−0.27* (0.020)	−0.31, −0.23	−8.86
	12	−0.14* (0.007)	−0.15, −0.12	−41.28	−0.28* (0.027)	−0.34, −0.23	−13.06	−0.30* (0.021)	−0.34, −0.26	−10.32
	24	−0.16* (0.010)	−0.18, −0.14	−47.80	−0.42* (0.026)	−0.47, −0.37	−19.42	−0.45* (0.028)	−0.50, −0.40	−15.33
Overall lingual	6	−0.13* (0.009)	−0.15, −0.11	−28.74	−0.27* (0.022)	−0.31, −0.22	−13.71	−0.25* (0.020)	−0.29, −0.21	−7.72
	12	−0.14* (0.009)	−0.16, −0.13	−38.30	−0.24* (0.029)	−0.30, −0.19	−11.01	−0.30* (0.021)	−0.34, −0.26	−9.62
	24	−0.18* (0.012)	−0.20, −0.15	−44.05	−0.41* (0.029)	−0.46, −0.35	−18.07	−0.38* (0.027)	−0.43, −0.33	−12.71
Overall facial	6	−0.11* (0.007)	−0.13, −0.10	−33.69	−0.32* (0.021)	−0.37, −0.28	−17.28	−0.29* (0.027)	−0.34, −0.23	−10.10
	12	−0.13* (0.007)	−0.14, −0.11	−45.26	−0.32* (0.028)	−0.38, −0.27	−15.27	−0.30* (0.028)	−0.36, −0.25	−11.06
	24	−0.15* (0.011)	−0.17, −0.13	−53.67	−0.44* (0.027)	−0.50, −0.39	−20.91	−0.52* (0.038)	−0.60, −0.45	−18.09
Margin/body overall	6	−0.11* (0.007)	−0.12, −0.09	−27.45	−0.33* (0.023)	−0.38, −0.29	−18.08	−0.28* (0.024)	−0.33, −0.24	−9.90
	12	−0.12* (0.007)	−0.13, −0.11	−38.22	−0.32* (0.030)	−0.38, −0.26	−15.71	−0.32* (0.026)	−0.37, −0.27	−11.46
	24	−0.14* (0.011)	−0.16, −0.12	−47.05	−0.48* (0.030)	−0.54, −0.42	−23.00	−0.50* (0.037)	−0.58, −0.43	−18.90
Margin/body lingual	6	−0.12* (0.010)	−0.14, −0.10	−24.36	−0.30* (0.025)	−0.35, −0.25	−15.85	−0.26* (0.023)	−0.30, −0.21	−8.27
	12	−0.13* (0.010)	−0.15, −0.11	−34.93	−0.27* (0.032)	−0.33, −0.20	−12.56	−0.33* (0.025)	−0.38, −0.28	−10.77
	24	−0.15* (0.014)	−0.18, −0.12	−42.80	−0.44* (0.032)	−0.50, −0.37	−20.34	−0.43* (0.035)	−0.50, −0.36	−15.25
Margin/body facial	6	−0.10* (0.007)	−0.11, −0.08	−32.20	−0.36* (0.026)	−0.41, −0.31	−20.51	−0.31* (0.034)	−0.37, −0.24	−11.90
	12	−0.11* (0.007)	−0.12, −0.09	−43.50	−0.38* (0.033)	−0.45, −0.31	−19.10	−0.30* (0.036)	−0.37, −0.23	−12.32
	24	−0.13* (0.012)	−0.16, −0.11	−53.10	−0.52* (0.033)	−0.58, −0.46	−25.95	−0.58* (0.052)	−0.68, −0.48	−23.08
Papillae/interproximal overall	6	−0.14* (0.008)	−0.15, −0.12	−34.53	−0.26 (0.019)	−0.30, −0.22	−12.91	−0.26* (0.019)	−0.30, −0.22	−8.38
	12	−0.15* (0.008)	−0.17, −0.14	−44.33	−0.24 (0.026)	−0.29, −0.19	−10.58	−0.30* (0.020)	−0.33, −0.26	−9.80
	24	−0.19* (0.012)	−0.21, −0.17	−48.62	−0.37 (0.025)	−0.41, −0.32	−15.94	−0.42* (0.025)	−0.47, −0.37	−13.77
Papillae/interproximal lingual	6	−0.15* (0.010)	−0.17, −0.13	−33.95	−0.24 (0.023)	−0.28, −0.19	−11.68	−0.24* (0.019)	−0.28, −0.20	−7.43
	12	−0.15* (0.010)	−0.17, −0.14	−41.97	−0.22 (0.029)	−0.28, −0.17	−9.59	0.29* (0.020)	−0.33, −0.25	−9.03
	24	−0.21* (0.016)	−0.24, −0.18	−45.40	−0.38 (0.028)	−0.43, −0.32	−15.94	−0.35* (0.024)	−0.40, −0.31	−11.48
Papillae/interproximal facial	6	−0.13* (0.009)	−0.15, −0.11	−35.06	−0.28 (0.020)	−0.32, −0.24	−14.26	−0.28* (0.025)	−0.32, −0.23	−9.31
	12	−0.15* (0.009)	−0.17, −0.13	−46.91	−0.26 (0.027)	−0.32, −0.21	−11.67	−0.30* (0.026)	−0.35, −0.25	−10.53
	24	−0.17* (0.014)	−0.19, −0.14	−53.72	−0.36 (0.026)	−0.41, −0.31	−16.00	−0.49* (0.035)	−0.56, −0.42	−16.03

*Note*: % Diffe = 100* (Adjusted mean in 67% sodium bicarbonate – adjusted mean in negative)/(Adjusted mean in negative control).

Abbreviations: CI, confidence interval, % Diff, percentage difference, SE, standard error.

* *p* < 0.0001.

Analysis by tooth region revealed that for all regions, statistically significant (*p* < 0.0001) differences in favour of 67% sodium bicarbonate toothpaste compared with the control were observed, also with progressive improvements at each time point. Comparison between regions showed that facial regions provided numerically greater between treatment improvements (% difference in favour of 67% sodium bicarbonate) than lingual regions for all measures at all post‐treatment time points. Margin/body regions (overall, lingual and facial) provided numerically greater between treatment improvements than papillae/interproximal regions for MGI and TPI, respectively, at all timepoints. However, for BS/BI, papillae (overall, lingual and facial) regions showed slightly greater between treatment improvements than margin areas. The greatest between treatment improvements (% difference in favour of 67% sodium bicarbonate) were seen for the papillae facial areas for BS (−54.16%)/BI (−53.72%) and margin/body facial for MGI and TPI (−25.95 and −23.08, respectively) at 24 weeks.

**FIGURE 1:**
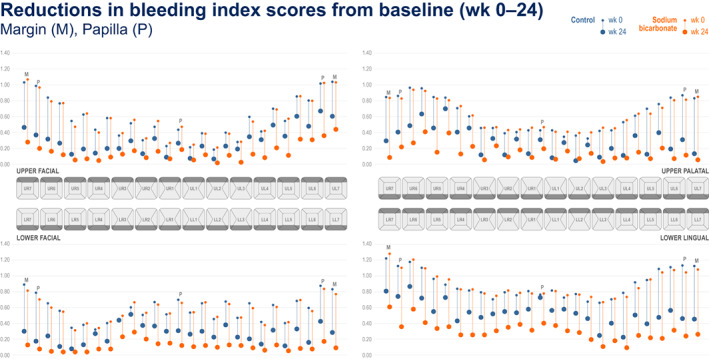
Bleeding index score at baseline and 24 weeks by tooth site for the 67% sodium bicarbonate toothpaste and control toothpaste.

**FIGURE 2:**
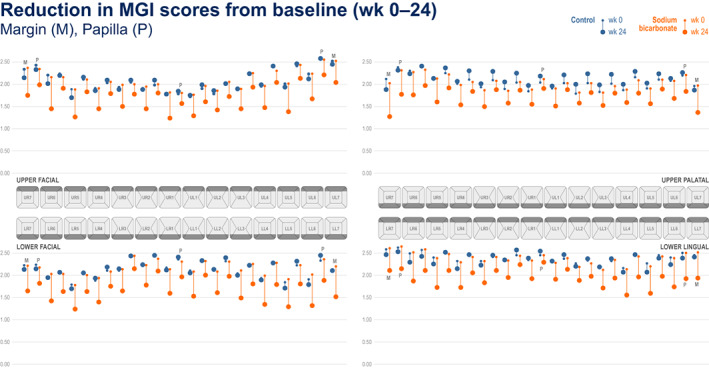
Modified gingival index score at baseline and 24 weeks by tooth site for the 67% sodium bicarbonate toothpaste and control toothpaste.

**FIGURE 3:**
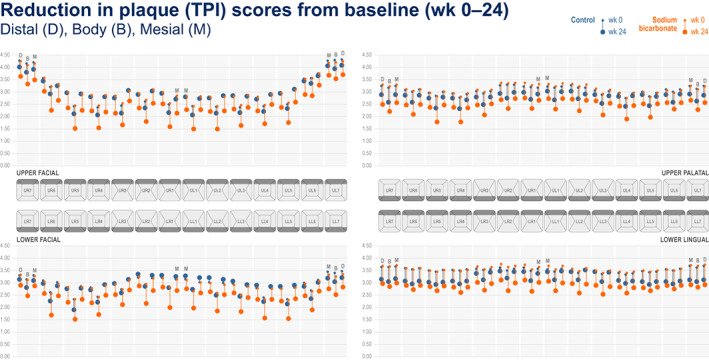
TPI score at baseline and 24 weeks by tooth site for the 67% sodium bicarbonate toothpaste and control toothpaste.

Figures 1, 2, 3 present ‘mouth maps’ depicting reductions from baseline for measures of gingival health and plaque for 67% sodium bicarbonate and control toothpastes by tooth site, at week 24 (BI (Figure 1), MGI (Figure 2) and TPI (Figure 3)). For all measures at all tooth sites, numerically greater reductions from baseline were observed for the 67% sodium bicarbonate toothpaste compared with the control. For BI, highest baseline scores were seen in the posterior teeth and for papillae sites (notably in the facial regions). For all regions and treatments, generally the largest reductions from baseline following treatment were observed in papillae sites, with the exception the upper palatal region where margin sites showed greater reduction from baseline than papillae sites. For MGI, the baseline scores were generally consistent across all regions although slightly higher baseline scores were observed for the posterior teeth (tooth numbers 6 and 7) in the upper facial region. For MGI, the highest baseline scores were seen in the papilla sites (compared with the equivalent margin tooth site) in all regions, however in contrast to BI, the greatest reductions from baseline were generally observed for the margin sites. For TPI, elevated baseline scores were observed for the posterior teeth (tooth numbers 6 and 7), most notably for the facial region. Body sites generally demonstrated the lowest baseline scores, and the greatest change from baseline was observed in the body sites.

It was observed that the improvement in all gingivitis variables (number of bleeding sites, BI, and MGI) at all relevant time points for relevant treatment groups was consistent across countries (USA and India). Similarly, the improvement in plaque variable (TPI) at all relevant time points for relevant treatment groups was also consistent across countries (China, India, and USA).

### 3.3 Safety

This pooled analysis of individual patient level data focused on the efficacy of twice‐daily use of a 67% w/w sodium bicarbonate toothpaste, with no study specific objective to pool the safety data across the studies. All the products were generally well tolerated in the clinical studies. Further details of the adverse events reported within the individual studies are provided in the supplementary data Table S3.

## 4 DISCUSSION

To the authors’ knowledge, this is the first pooled analysis of individual patient level data from similarly designed clinical studies investigating the gingival health benefits of a 67% sodium bicarbonate toothpaste. Pooled analysis of these six studies, conducted across three countries involving 1474 subjects demonstrates large and statistically significant improvements in measures of gingivitis (number of bleeding sites, BI, and MGI); a greater than 15% difference between the 67% w/w sodium bicarbonate toothpaste and the negative control was observed for the measures of gingival health. Notably, over the 24‐week treatment period, the number of bleeding sites per subject reduced from a mean of 56 to a mean of 16 bleeding sites in the 67% sodium bicarbonate toothpaste treatment group. Expressed in terms of proportion (%) with respect to the number sites assessed (128/subject), this represents a reduction from 44% bleeding sites at baseline to approximately 12% after 24 weeks twice daily use; which could be described as a shift from “generalized” gingivitis (≥30% sites of the teeth affected by gingival inflammation) or “localized” gingivitis (<30% sites) gingivitis” to “incipient gingivitis” (only a few sites affected by mild inflammation).^20^ These results reinforce the clinically relevant long‐term effect of twice daily use of 67% sodium bicarbonate toothpaste in reducing plaque build‐up and gingivitis, following a professional dental prophylaxis and continued twice daily use.

The tooth site analysis demonstrated that at all timepoints the plaque scores for the body sites demonstrated greater improvements (% difference between treatments) than interproximal sites for the 67% sodium bicarbonate toothpaste. This observation contrasts with that reported for single‐use plaque removal studies^14^ where a 67% sodium bicarbonate toothpaste demonstrated the greatest removal of plaque in the interproximal tooth site areas. However, this observation can be explained by considering the differences in the nature of plaque between single‐use plaque removal studies and long‐term gingival health studies (in subjects with pre‐existing gingivitis). Single‐use plaque removal studies generally include ‘high plaque formers’ (typical TPI >2.5 inclusion criteria) with no/low underlying gingivitis^14^, ^15^, ^16^, ^17^, ^18^, ^19^, ^20^, ^21^ and explore the removal of immature plaque (plaque from subjects who have been asked to obtain from oral hygiene procedures for 24 h), to provide understanding on the mode of action of formulations.^14^ The nature of plaque in single use studies is typically thin, of low complexity and less resistant to removal than mature plaque that has been allowed to accumulate in the ‘sheltered’ areas. In contrast, long‐term oral hygiene and gingival health studies generally explore removal of plaque with a range of maturity. Nevertheless, in this pooled analysis of long‐term plaque and gingivitis studies, while the plaque reduction in the interproximal sites was not the largest out of all the areas explored, it did result in the greatest improvements (% difference compared with control) in gingival bleeding, demonstrating that the nature/maturity of plaque removed is an important factor for people prone to gingivitis, and that even small reductions in mature plaque (in the interproximal areas) can provide significant improvements in gingival health.

This pooled analysis also demonstrated that improvements in all clinical measures of gingival health and oral hygiene were consistent across the three countries in which the individual studies were conducted.

An advantage of this pooled analysis is that it employed studies of very similar design, which provided reliable information with reduced heterogeneity, to explore plaque control and gingival health benefits of a 67% sodium bicarbonate toothpaste at the individual tooth site level. However, equally this could be seen as a limitation, and the results should only be viewed in the context of patients with gingivitis (no history of periodontitis) and who undergo/have access to an appropriate standard of dental care (regular professional cleaning), and maintain long‐term compliance with maintain oral hygiene. This pooled analysis also only included studies in which the Investigators had access to patient‐level data (studies conducted by GSKCH/Haleon) in order to conduct a tooth site analysis. This study could be improved by conducting a broader analysis using patient‐level data from studies conducted by other institutions. Anonymized individual participant data and study documents from this study can be requested for further research from www.clinicalstudydatarequest.com.

## 5 CONCLUSION

This pooled analysis of patient‐level data from six similarly designed studies demonstrates gingival health and plaque control in participants with mild to moderate gingivitis were significantly improved following a professional dental clean and twice‐daily brushing with a 67% sodium bicarbonate toothpaste compared with a regular (non‐sodium bicarbonate) toothpaste. Improvements in the measure of gingival health and oral hygiene were consistent across all tooth sites suggesting that the mechanical action of sodium bicarbonate (toothpastes) extends to all tooth sites.

## 6 CLINICAL RELEVANCE

As a supplement to professional dental cleaning, the long‐term use of a sodium bicarbonate (67%) toothpaste twice daily is more effective at removing plaque biofilms and improving gingival health (at all tooth sites in the mouth) compared with a non‐sodium bicarbonate toothpaste.

## AUTHOR CONTRIBUTIONS

CP contributed to conception, design and interpretation of the study and results and drafted the manuscript. AB and ML contributed to conception, design and interpretation of the study and results and critically reviewed the manuscript.

## FUNDING INFORMATION

This study was funded by Haleon (previously GSK Consumer Healthcare).

## CONFLICT OF INTEREST

CP, AB and ML are employees of Haleon, who provided funding for this study.

## Supporting information


Tables S1–S3

## Data Availability

The data that support the findings of this study are available on request from www.clinicalstudydatarequest.com.
