# Supplementary material for: Antigingivitis efficacy of a sodium bicarbonate toothpaste: Pooled analysis
Source: Int J Dent Hyg. 2022 Oct 5;21(1):106–15. doi: 10.1111/idh.12626 (PMC10092887; doi:10.1111/idh.12626)
Supplement: Supplementary file 1 — Tables S1–S3 [file IDH-21-106-s001.docx]

**Supplementary Data**

**Table 1: Studies identified and reviewed for inclusion in this pooled analysis.**

| Study Number | Length of the Study (Time Points) | Clinical Efficacy Measures | Study Design | Included in Pooled Analysis | Main Reason for Exclusion |
| --- | --- | --- | --- | --- | --- |
| E5931015; In  (Bosma et al 2018) | Single- brushing* | TPI | Crossover | No | No  pre-prophylaxis |
| E5930966; In  (Bosma et al 2018) | Single- brushing* | TPI | Crossover | No | No  pre-prophylaxis |
| 202655 (RH01455);  In (Bosma et al 2018) | Single- brushing* | TPI | Crossover | No | No  pre-prophylaxis |
| 202644 (RH01472);  In (Kakar et al 2014) | Baseline, 6,  and 12 weeks | Number of bleeding sites, mean BI, TPI | Parallel | No | No  pre-prophylaxis |
| 202694 (RH01530);  In (Kakar et al 2014) | Baseline, 6,  and 12 weeks | Number of  bleeding sites, mean BI, TPI | Parallel | Yes | - |
| 202806 (RH01750);  In (Newby et al 2014) | Baseline, 6,  and 12 weeks | GSI, GI, TPI | Parallel | Yes | - |
| 202812 (RH01763);  In (Kakar et al 2014) | Baseline, 6,  and 12 weeks | Number of  bleeding sites, mean BI, TPI | Parallel | Yes | - |
| 202779 (RH01697);  Unpublished | Baseline, 1,  and 2 weeks | Oral tolerability* | Parallel | No | No efficacy endpoint |
| 202388 (RH01977);  In (Lomax et al 2017) | Baseline and 6 weeks | Number of bleeding sites, mean BI, MGI,  TPI | Parallel | Yes | - |
| 202193 (RH02434);  In (Jose et al 2018) | Baseline, 6,  12, and 24 weeks | Number of bleeding sites, MGI, TPI | Parallel | Yes | - |
| 202192 (RH02433);  In (Akwagyiram et al 2018) | Baseline, 6,  12, and 24 weeks | Number of bleeding sites, MGI, TPI | Parallel | Yes | - |
| 203067 (RH02683);  Unpublished | Single-rinse* | TPI | Crossover | No | No  pre-prophylaxis |
| 206886;  Unpublished | Single- brushing* | TPI | Crossover | No | No  pre-prophylaxis |

* Reason for exclusion based on pre-defined study selection criteria

Abbreviations: BI = Bleeding Index, GI = Gingival Index, GSI = Gingival Severity Index, MGI = Modified Gingival Index, TPI = Turesky Plaque Index.

# Table 2 Overview of studies selected for inclusion in the pooled analysis

| Reference Number | | Kakar,  2014  202694 | Newby, 2014  202806 | Kakar, 2014  202812 | Lomax, 2017  202388 | Akwagyirama 2017  202193 | Jose, 2018  202192 |
| --- | --- | --- | --- | --- | --- | --- | --- |
| Design | | Single-center, examiner-blind, parallel group, stratified, randomized | | | | | |
| Prophylaxis at | | Baseline | Baseline | Baseline | Baseline | Screening | Screening |
| Study period (FSFV-LSLV) | | Jul 2012 –  Nov 2012 | May 2013 –  Aug 2013 | Jun 2013 –  Oct 2013 | Nov 2013 –  Jan 2014 | Aug 2014 –  Apr 2015 | Aug 2014 –  Mar 2015 |
| Treatment group | Test product(s) | 67% and 62% w/w sodium bicarbonate | | | 67% w/w sodium bicarbonate | | |
|  | Control product | Regular toothpaste (0% sodium bicarbonate) | | | | | |
| Product application/ dosing | | Subjects brushed teeth with a full strip of toothpaste covering the entire head of the toothbrush for 1 minute twice a day – morning and evening | | | | | |
| Time points for efficacy assessments | | Baseline, Weeks 6, and 12 | | | Baseline, Week 6 | Baseline, Weeks 6, 12,  and 24 | |
| Endpoints | Primary | Mean no. bleeding sites (4 sites)  mean GSI (6 sites) | | | Mean no. bleeding sites (4 sites) | Mean no. bleeding sites and mean MGI (4 sites) | |
|  | Secondary | Mean no. bleeding sites Mean BI, Mean GSI Mean TPI | | | Mean BI  Mean MGI  and VSC | Mean no. bleeding sites Mean MGI  Mean BI Mean TPI | |
| Study location | | India | China | India | India | USA | USA |
| No. randomized subjects (N) | | 330 | 342 | 288 | 148 | 246 | 247 |
| ITT Population (N) | | 309 | 336 | 279 | 135 | 235 | 240 |
| Gender (% male / female) | | 51/ 49% | 25/ 75% | 51/ 49% | 43/ 57% | 38/ 62% | 43/ 57% |
| Stratification | | Baseline no. bleeding sites and smoking status | | | | | |

Abbreviations: BI = Bleeding Index, FSFV = first subject first visit, GSI = Gingival Severity Index, ITT = intent-to-treat, LSLV = last subject last visit, MGI = Modified Gingival Index, N = number of subjects, no. = number of, TPI = Turesky Plaque Index, USA = United States of America, VSC = volatile sulphur compounds.

**Table 3: Adverse events (AEs) reported within the individual GSKCH clinical study reports.**

| **Study** | **67% sodium bicarbonate toothpaste** | | | **Negative control (0% sodium bicarbonate)** | | |
| --- | --- | --- | --- | --- | --- | --- |
|  | **Treatment Emergent AEs** | | **Treatment Emergent-Treatment Related AEs** | **Treatment Emergent AEs** | | **Treatment Emergent-Treatment Related AEs** |
|  | **All** | **Oral** |  | **All** | **Oral** |  |
| 202812 | **0** | **0** | **0** | **4** | **2** | **0** |
| 202806 | **4** | **2** | **0** | **3** | **2** | **0** |
| 202694 | **22** | **1** | **0** | **13** | **1** | **0** |
| 202388 | **2** | **1** | **0** | **2** | **2** | **0** |
| 202193 | **7** | **10**  **5** | **0** | **10** | **8** | **0** |
| 202192 | **22** | **10** | **2 (burning sensation (perioral); generalised tooth sensitivity)** | **16** | **11** | **5 (diarrhoea, thermal burn, tooth sensitivity, angular cheilitis (x2).** |
